# Lower ureteral compression through external vascular elongation in a cyclist: A case report

**DOI:** 10.1016/j.ijscr.2021.106031

**Published:** 2021-05-26

**Authors:** Christian Ramesmayer, Michael Mitterberger, Lukas Oberhammer, Thomas Kunit, Lukas Lusuardi

**Affiliations:** Department of Urology and Andrology, Paracelsus Medical University, Salzburg, Austria

**Keywords:** Lower ureteric obstruction, Crossing vessel, Ureterocystoneostomy, Vascular elongation, Case report

## Abstract

**Introduction and importance:**

There is sparse literature about lower ureteric obstruction due to aberrant blood vessels. We report a case of a patient who was referred to our hospital due to left sided flank pain caused by external compression of the distal ureter.

**Case presentation:**

A 47-year-old male patient presented with left sided flank pain. A computed tomography scan revealed external compression of the lower ureter. Hypertrophy of the psoas muscle due to extensive cycling for 20 years lead to concomitant kinking and elongation of the iliacal vessels which caused the distal ureteric obstruction. Robotic-assisted laparoscopic ureterocystoneostomy with psoas hitch technique was performed.

**Clinical discussion:**

Lower ureteric obstruction, mostly seen in children, is mostly caused by vascular anomalies such as a persistent umbilical artery. After literature review, we presume it to be the first reported case of distal ureteric obstruction caused by external vascular elongation.

**Conclusion:**

The external elongation of pelvic vessels due to excessive cycling and the concomitant extrinsic compression of the distal ureter should be considered as rare but possible cause of lower uretic obstructions.

## Highlights

• A 47-year-old cyclist was admitted with left sided flank pain
• Excessive cycling lead to elongation of the iliacal vessels and concomitant hydronephrosis
• External compression of distal ureter due to pelvic elongation is a rare differential diagnosis of lower ureteric obstruction
• Robotic assisted ureterocystoneostomy as a treatment option was performed

## 1 Introduction

Ureteral obstruction can happen due to many different causes (intrinsic or extrinsic compression). Ureter stones and malignancies are common reasons for obstruction. Undetected injuries after abdominal surgery as well as traumatic causes are also known etiologies for ureteric stricture. Rare causes are infectious diseases such as tuberculosis or ureteric lymphoma [1]. Lower ureteral obstruction is less frequently reported than obstruction in the upper part. Abdominal and iliacal aneurysms which lead to ureteral fibrosis are also documented in the literature as rare causes. There are only a few reports in literature about distal ureteric obstruction due to vascular abnormalities such as persistent umbilical artery or ligament. We are reporting a rare case of distal ureteric obstruction due to elongation and kinking of the pelvic vessels caused by psoas hypertrophy in a cyclist, which we presume to be the first of its kind after literature review. This work has been reported in line with the 2020 SCARE criteria [2].

## 2 Case presentation

**Fig. 1:**
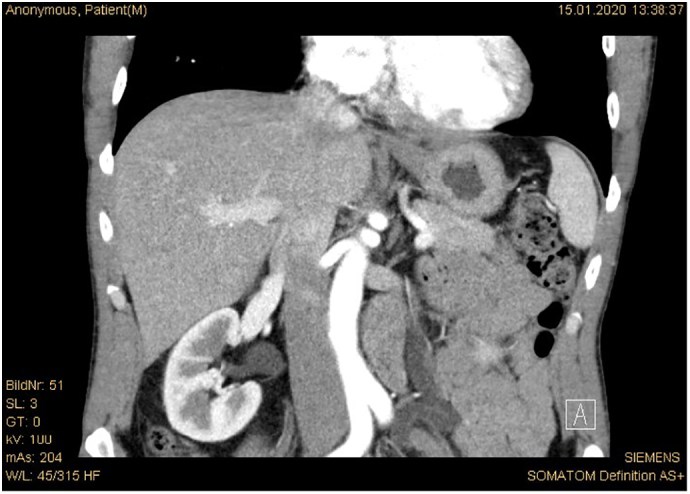
Contrast-enhanced computed tomography scan (arterial phase) showing slightly enlarged common iliac artery with kinking and concomitant secondary hydronephrosis.

A 47-year-old male patient was referred to our department from the general practitioner with recurrent left sided flank pain for 3 months. He had no urological history and his serum creatinine level was normal. A previous abdominal ultrasound revealed left sided secondary hydronephrosis, kinking of the iliac artery and probably a neoplasm of the ureter. Hence, a contrast-enhanced computed tomography scan was performed, which showed an extrinsic compression of the left ureter at the level of the bifurcation of the common iliac artery (Fig. 1). No intra-abdominal aneurysms were detected.

**Fig. 2:**
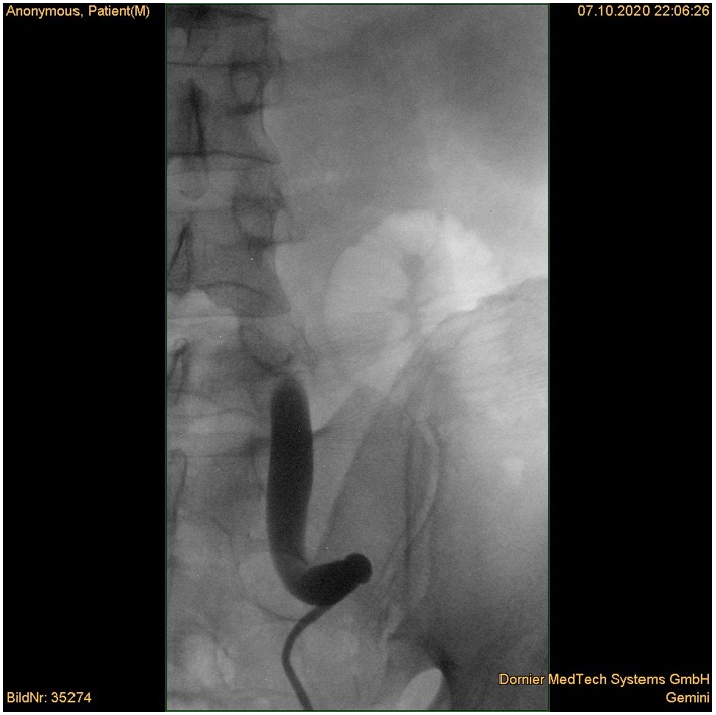
Retrograde Ureteropyelography showing ureteral kinking and distal obstruction.

Afterwards the patient underwent retrograde pyelography and primary ureterorenoscopy with double-J stenting, which revealed the ureteric stricture at the level of the bifurcation of the common iliac artery (Fig. 2). During the ureterorenoscopy the pulsation of the vessel was seen in the stenotic area and histological samples were taken from the region of interest. Histopathology of the resected ureteric segment showed slight chronic inflammation of the ureter.

Afterwards, the patient was presented to the colleagues from the vascular surgery in our hospital. The patient had no symptoms concerning angiopathy especially no signs of intermittent claudication. He has been a cyclist for over 20 years with about 10,000 driven kilometers per year.

In the clinical examination all pulses (femoral, popliteal, posterior tibial) were well palpated. After re-evaluation of the computed tomography scan, hypertrophy of the psoas muscle as well as extension of the internal iliac artery (diameter 1.0 × 1.1 cm) could be shown. In men, the average diameter of the iliac artery is 1.2 ± 0.2 cm.

**Fig. 3:**
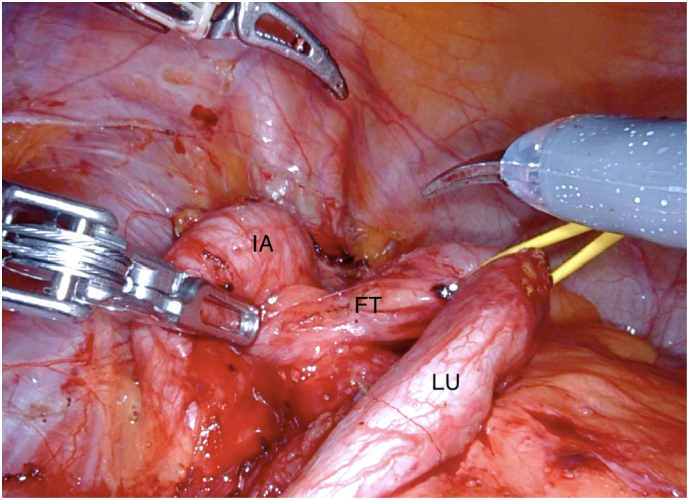
Intraoperative picture showing compression of the lower ureter (LU) caused by arterial kinking of the iliac artery (IA). The LU is connected with the artery by fibrous tissue (FT).

In our view, the muscular hypertrophy could be explained by the strong hip flexion due to extensive cycling for several years and the hypertrophy caused the concomitant enlargement and kinking of the iliacal vessels. Especially the artery was made responsible for the lower ureteral obstruction. After interdisciplinary discussion with the vascular surgeons, no vessel reconstruction was recommended. The patient was admitted to robotic-assisted laparoscopic ureterocystoneostomy. The stenotic area, which could be seen directly next to the artery, was resected and the patient underwent ureterocystoneostomy with psoas hitch technique. The histopathological samples showed no malignancy (Fig. 3). The patient recovered well.

## 3 Discussion

The etiologies of ureteric strictures are heterogeneous. Upper ureteric obstruction by aberrant vessels is a well-documented pathology, whereas literature about lower ureteric compression by blood vessels is rare. In 1929, Hyams reported the first time of an extrinsic obstruction of the lower ureter due to an aberrant vessel [3].

Five cases of anomalous arteries leading to ureter compression were presented by Young and Kiser in 1965 [4]. In these cases the authors performed ureteroureto(cystoneo)stomy to excise the obstruction instead of resecting the vessel.

Obstruction of the distal ureter occurs more often during childhood. Read and Devine reported of 7 distal ureter obstructions in children up to 6 years of age caused by vascular anomalies [5]. Four of them were treated with ureterocystoneostomy, the other three patients underwent vessel transection. In 1997 Kurimoto et al. showed a case of a child with secondary hydronephrosis due to an aberrant vessel originating from the iliac artery. The boy was treated with an end-to-end anastomosis of the ureter [6]. Quattlebaum and colleagues reported of a similar case in an adult. The 79-year-old patient underwent ureteroureterostomy and recovered well [7].

In 1992 Grifoni et al. summarized 11 cases of ureteric obstruction caused by anatomical variants of the umbilical artery. According to the authors anomalies of the umbilical artery are rare but possible causes of lower ureteral obstruction [8].

Gupta et al. presented a case of a 32-year-old woman with persistent umbilical artery leading to obstruction of the ureter. Interestingly, the patient was treated with ureterocystoneostomy and resection of the vessel [9].

The latest report to our knowledge is also the first published case, where distal ureteric obstruction was caused by an aberrant gonadal vein and not by an artery. Raghavendran et al. resected the stenotic ureteric segment and performed ureterocystoneostomy. No clinical symptoms were reported in the one-year follow-up [10].

## 4 Conclusion

The external elongation of pelvic vessels due to excessive cycling and the concomitant extrinsic compression of the distal ureter should be considered as rare but possible cause of lower uretic obstructions. Ureterocystoneostomy may be an option for definitive treatment.

## Consent

Written informed consent was obtained from the patient for publication of this case report and accompanying images. A copy of the written consent is available for review by the Editor-in-Chief of this journal on request.

## Provenance and peer review

Not commissioned, externally peer-reviewed

## Ethical approval

Not applicable

## Funding

This study has not received funding from any person or institution.

## Guarantor

Christian Ramesmayer is the guarantor and accepts full responsibility for the work and/or the conduct of the study, had access to the data, and controlled the decision to publish.

## Research registration number

Not applicable.

## CRediT authorship contribution statement

Christian Ramesmayer, Michael Mitterberger and Lukas Lusuardi designed and directed the project. Christian Ramesmayer took the lead in writing the manuscript. All authors provided critical feedback and helped shape the research, analysis and manuscript.

## Declaration of competing interest

The authors report no declaration of interest.
